# Morphologic Features of Cutaneous T-Cell Lymphomas Using Dermoscopy and High Frequency Ultrasound

**DOI:** 10.3390/jcm10010017

**Published:** 2020-12-23

**Authors:** Iris Wohlmuth-Wieser, Joel M. Ramjist, Neil Shear, Raed Alhusayen

**Affiliations:** 1 Division of Dermatology, Department of Medicine, Sunnybrook Health Sciences Center, Toronto, ON M4N 3M5, Canada; neil.shear@sunnybrook.ca (N.S.); raed.alhusayen@sunnybrook.ca (R.A.); 2 Department of Medicine, University of Toronto, Toronto, ON M5S 1A8, Canada; 3 Department of Dermatology and Allergology, Paracelsus Medical University Salzburg, 5020 Salzburg, Austria; 4 Department of Electrical, Computer, and Biomedical Engineering, Ryerson University, Toronto, ON M5B 2K3, Canada; j.ramjist@gmail.com

**Keywords:** cutaneous T-cell lymphoma, mycosis fungoides, dermoscopy, high frequency ultrasound, validation, atopic dermatitis, psoriasis

## Abstract

The diagnosis of cutaneous T-cell lymphomas (CTCL) is frequently delayed by a median of three years and requires the clinical evaluation of an experienced dermatologist and a confirmatory skin biopsy. Dermoscopy and high-frequency ultrasound (HFUS) represent two non-invasive diagnostic tools. While dermoscopy is inexpensive and widely used for the diagnosis of melanoma and non-melanoma skin cancers, HFUS of skin lymphomas represents a novel diagnostic approach that is not yet implemented in the routine dermatologic practice. The aim of our study was to prospectively assess skin lesions of patients with either CTCL patches or plaques with dermoscopy and HFUS and to compare the findings with atopic dermatitis (AD) and psoriasis. Thirteen patients with an established diagnosis of CTCL, psoriasis, or AD were studied: Dermoscopy features including spermatozoa-like structures and the presence of white scales could assist in differentiating between early-stage CTCL and AD. HFUS measurements of the skin thickness indicated increased epidermal-, thickness in CTCL, and psoriasis compared with AD. Our results support the use of dermoscopy as a useful tool to diagnose CTCL. HFUS could augment the dermatologic assessment, but further studies will be needed to define standardized parameters.

## 1. Introduction

Cutaneous T-cell lymphomas (CTCL) represent a heterogeneous group of Non-Hodgkin lymphomas that are characterized by neoplastic T lymphocytes infiltrating the skin. Primary CTCL show no evidence of extra-cutaneous manifestation at diagnosis, with Mycosis fungoides (MF) being the most prominent representative [1]. The estimated annual incidence of CTCL is 10.2 per million persons, with an increased incidence in the fifth decade of life [2]. Clinically, MF is characterized by the presence of red scaly patches or plaques, predominantly on sun-protected areas of the body [3]. In later disease stages, patients may develop thick infiltrated plaques, tumours, or leukemic disease with lymph node and visceral involvement [3,4]. Due to its variable clinical presentation, differentiation from inflammatory skin diseases can be challenging. Therefore, MF has been described as one of the great dermatological imitators [5], requiring experienced clinicians and a sophisticated interplay between clinical evaluation, histopathology, and molecular biology to establish the correct diagnosis [6]. Recent prospectively acquired data show that MF is associated with a diagnostic delay between first symptom development and final diagnosis of three years [6]. 

In many cases, more than one biopsy and several consultations are needed to differentiate the disease from other benign inflammatory conditions [6]. Over the last years dermatologists have been seeking alternative diagnostic tools to facilitate the diagnosis of CTCL [7,8]. 

Dermoscopy is an inexpensive, non-invasive method that is commonly used for the assessment of melanocytic and non-melanocytic skin neoplasms [9]. Its application has been broadened to inflammatory skin diseases and recently to aid the diagnosis of CTCL [7,10]. High-frequency ultrasonography (HFUS) represents another non-invasive method used to assess skin neoplasms. Preliminary results support its applicability in the diagnosis of MF [11,12]. 

The aim of this study was to prospectively assess CTCL patients using dermoscopy and HFUS and compare the acquired features with patients with atopic dermatitis (AD) and psoriasis. 

## 2. Material and Methods

### 2.1. Patients

We prospectively recruited 13 patients, with an established diagnosis of either CTCL, psoriasis, or AD. The study was conducted at the Division of Dermatology, Department of Medicine, Sunnybrook Health Sciences Center, Toronto, Canada, and was approved by the institutional review board (REB#299-2019). Prior to patient recruitment, the study was registered in the International Standard Randomised Controlled Trial Number (ISRCTN) registry (ISRCTN14038061). Patients were identified either at the routine dermatology clinic or at the multidisciplinary CTCL clinic. The diagnosis of CTCL was made according to the World Health Organization and European Organisation for the Research and Treatment of Cancer classification [1] and was confirmed by skin biopsy in all included patients. 

Patients were then invited to a dedicated research clinic. Written informed consent was obtained from every individual participant prior to study inclusion. A representative skin lesion (either patch in CTCL/AD or plaque in CTCL/psoriasis patients) was determined and clinical photographs, dermoscopy images, and an HFUS scan were obtained for further analyses. The location of the acquired image was documented, and skin types were assessed using the Fitzpatrick Scale. 

### 2.2. Methods

#### 2.2.1. Dermoscopy 

Dermoscopic images were taken of a representative skin lesion, using a DermLite DL4^®^ (3Gen Inc., San Juan Capistrano, CA, USA) with 10-fold magnification. The images were stored digitally, and the following criteria were analyzed: vascular pattern (fine short linear vessels, dotted vessels, spermatozoa-like structures, pseudopod-like vessels, arborizing vessels, polymorphous vascular pattern), background color (light red background, dull red background), scales (white scale, yellow scale), structureless patches, orange-yellowish patchy areas, crystalline structures, yellow ulceration, perifollicular accentuation, and comedo openings. We further compared our results with data retrieved from previously published studies, identified through a literature search [10,13,14]. 

#### 2.2.2. High-Frequency Ultrasound

Skin ultrasonography was performed in vivo from the index lesion using a Vevo MD^®^ (FUJIFILM VisualSonics, Toronto, ON, Canada) high-frequency ultrasound system. Cross-sectional skin images (B-mode) were obtained using a 70 MHz transducer with a central wavelength of 50 MHz (UHF70, FUJIFILM VisualSonics, Toronto, ON, Canada). To allow acoustic coupling, a conventional ultrasound gel was used to create a thin film and the axis of the transducer was placed perpendicular to the skin surface. 

The following parameters were measured in µm: epidermal thickness, thickness of the subepidermal low echogenic band (SLEB), dermal thickness. The epidermal thickness was measured from the epidermal entrance echo to the upper border of the SLEB. The SLEB was defined as a clearly visual low-echogenic band directly below the epidermis and was measured from the lower border of the epidermis to the upper dermal border. The dermal thickness was measured from the lower border of the epidermis or if present from the lower border of the SLEB to the interface of the dermis and hypodermis. Per patient, five measurements were taken for each parameter and the mean thickness was calculated. The SLEB was further graded as follows: grade 0, no visible SLEB; grade 1, subepidermal echolucent spots; grade 2 subepidermal echolucent patches; grade 3, continuous well-defined SLEB [15]. Additional morphologic parameters were documented for every analyzed image: evenness of the epidermis (even/uneven) and SLEB (even/uneven) and presence of dermal hypoechogenic foci. 

### 2.3. Statistics

All analyses were conducted using SPSS version 26 (IBM Corporation, Armonk, NY, USA). Continuous data are expressed as mean ± standard deviation (SD) or median (range), as appropriate. Continuous variables were compared using the Mann–Whitney test and categorical data were compared using the Fisher’s exact test. Sensitivity, specificity, positive predictive value (PPV) and negative predictive value (NPV) were calculated for dermoscopy features to distinguish patch stage CTCL from AD. 

A *p*-value < 0.05 was considered statistically significant.

## 3. Results

### 3.1. Demographic Data

A total of 13 patients (2 female and 11 males) were included in this study. Six of the 13 patients had CTCL, 5 psoriasis, and 2 AD. The stages of the CTCL patients were as follows: three patients with stage IB (patch only; two classic MF, one folliculotropic MF), one patient with stage IB (patch and plaque), and two patients with Sézary Syndrome (with well-defined patchy skin lesions). Skin types were assessed for every patient. The most common skin type screened was Type II (7/13), followed by Type I (2/13), Type III (2/13), and Type IV (2/13). Images were taken from the following body regions: arms (3/13), legs (5/13), and abdomen/flank (5/13). 

### 3.2. Dermoscopy

The dermoscopy features of all 13 patients were evaluated. Table 1 summarizes the dermoscopy findings of the CTCL patients including results from two previously published studies [10,13]. CTCL patches, CTCL plaques, and folliculotropic MF images were analyzed separately. Table 2 represents a pooled data analysis of observed features of our patient cohort (CTCL, AD, and Psoriasis) along with data retrieved from three studies [10,13,14] and compares the dermoscopy findings of CTCL patches with AD lesions and CTCL plaques with psoriasis plaques. 

**Figure 1:**
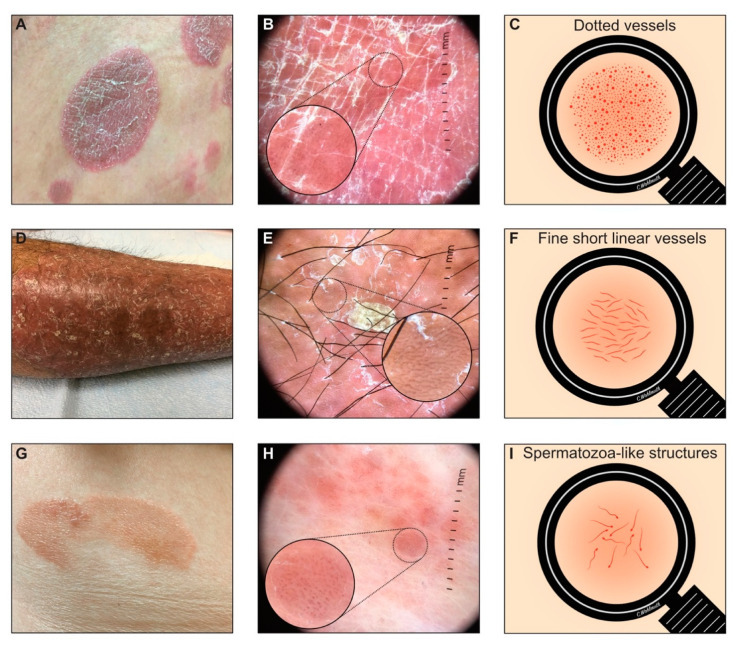
(**A**–**I**): Vascular patterns. Dermoscopic features of vascular patterns. (**A**–**C**): clinical, dermoscopy and schematic image of dotted vessels. (**D**–**F**): clinical, dermoscopy and schematic image of fine short linear vessels. (**G**–**I**): clinical, dermoscopy and schematic image of spermatozoa-like structures.

Commonly observed vascular patterns of CTCL patches include the presence of fine short linear vessels, dotted vessels and spermatozoa-like structures (Figure 1A–I and Table 1).

The presence of spermatozoa-like structures represents a highly specific dermoscopy feature for patch stage CTCL, compared to AD where none of the patients exhibited this feature (*p* < 0.001): The sensitivity, specificity, PPV and NPV to differentiate patch stage CTCL from AD were 52.4%, 100%, 100%, and 64.9%, respectively. Fine short linear vessels were observed in the majority of CTCL patches but only in one AD case (*p* < 0.001): The sensitivity, specificity, PPV, and NPV to differentiate patch stage CTCL from AD were 88.1%, 97.3%, 97.4%, and 87.8%, respectively. Dotted vessels, white scales and yellow scales were more common in AD. To distinguish AD from CTCL the sensitivity, specificity, PPV and NPV for dotted vessels were 86.5%, 50.0%, 60.4%, and 80.8%, respectively. 

For white scales, the sensitivity, specificity, PPV and NPV were 67.6%, 71.4%, 67.6%, and 71.4%, respectively, and for yellow scales the sensitivity, specificity, PPV, and NPV were 56.8%, 100%, 100%, and 72.4%, respectively.

The dermoscopy characteristics of CTCL plaques were assessed in two patients (one own patient and one patient published by Ghahramani et al. [13]). Both CTCL plaques showed dotted vessels on a dull red background with white scale (Table 1 and Table 2). Dotted vessels were also frequently found in psoriatic plaques, while none of the psoriatic patients had spermatozoa-like structures. 

### 3.3. High-Frequency Ultrasound 

**Figure 2:**
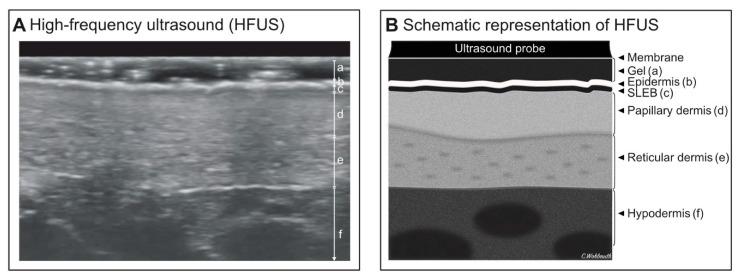
(**A**,**B**): Assessment of CTCL using HFUS. (**A**): High-frequency ultrasound image obtained in a CTCL patient depicting the epidermis (b), SLEB (c), papillary dermis (d), reticular dermis (e) and hypodermis/subcutis (f). (**B**): Schematic representation of the skin in high-frequency ultrasound images. Abbreviations: CTCL, cutaneous T-cell lymphoma; HFUS, high-frequency ultrasound; µm, micrometer; SLEB, subepidermal lower echogenic band.

**Figure 3:**
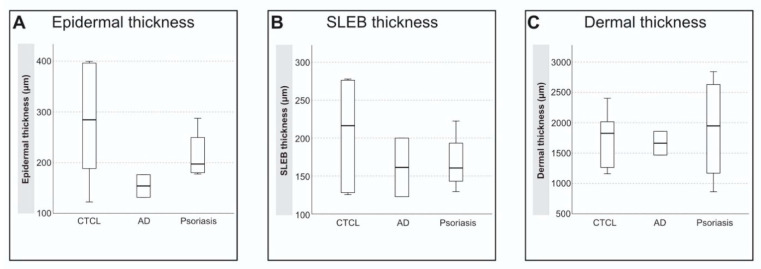
(**A**–**C**): Epidermal-, SLEB-, and Dermal thickness in CTLC, AD and Psoriasis. (**A**): Epidermal thickness in µm measured from the epidermal entrance echo to the upper border of the SLEB (average of 5 measurements per patient); (**B**): SLEB thickness in µm measured from the lower border of the epidermis to the upper dermal border (average of 5 measurements per patient); (**C**): dermal thickness in µm measured from the lower border of the epidermis or if present from the lower border of the SLEB to the interface of the dermis and hypodermis (average of 5 measurements per patient). Abbreviations: AD, atopic dermatitis; CTCL, cutaneous T-cell lymphoma; µm, micrometer; SLEB, subepidermal lower echogenic band.

The ultrasound measurements of 12 patients were analyzed (HFUS images could not be acquired in one patient due to technical difficulties on the day of the examination). Figure 2A,B shows an HFUS scan of a CTCL patient and a schematic representation of the skin in HFUS. All skin measurements are summarized in Table 3. The measurements of the mean epidermal-, SLEB-, and dermal thickness were compared within the three patient groups (CTCL, AD, and psoriasis). The mean epidermal thickness for CTCL patch lesions was 271 ± 124 µm, SLEB was observed in all CTCL patients, with grade 2 seen in (16.7%) and grade 3 in (83.3%). The mean SLEB thickness was 193 ± 78 µm and the mean dermal thickness was 1847 ± 460. Patients with CTCL and psoriasis tended to have an increased epidermal thickness compared to AD, although this was not significant in our limited sample size (Figure 3A–C). Patients with psoriasis had a slightly increased dermal thickness (1900 ± 897) compared with the CTCL patients (Figure 3C). 

Nine of the 12 patients (75%) had an uneven epidermis and all patients exhibited an uneven SLEB. Dermal hypoechogenic foci were observed in seven patients.

## 4. Discussion 

The diagnosis of CTCL is frequently delayed by three years [6]. Diagnosis is currently based on the clinical assessment and histopathologic evaluation of experienced dermatologists and pathologists [16]. Both dermoscopy and HFUS represent non-invasive diagnostic tools. While a hand-held dermatoscope is rather inexpensive and regularly used by dermatologists in all levels of training, HFUS of cutaneous lesions is currently not widely implemented in the dermatologic routine examination. Our study demonstrates the usefulness of additional diagnostic tools in the clinical setting and to facilitate the differentiation between CTCL lesions and inflammatory dermatosis.

### 4.1. Dermoscopy 

Our observed dermoscopy features support the findings of previous studies [10,13,17], indicating that certain vascular patterns are specific for early-stage MF patches. A combination of the two dermoscopy features—spermatozoa-like structures and white scale—could help differentiate an MF patch lesion from AD. Interestingly, our study and the cases published by Ghahramani et al. [13] did not observe the presence of orange-yellowish patchy areas, another feature that was previously described for patch stage CTCL [10]. 

Apart from the findings in classic CTCL patches, perifollicular accentuation, and comedo openings were clearly visible in folliculotropic MF patients. The implementation of dermoscopy in suspicious lesions may aid in the reduction of the diagnostic delay, but further large-scale studies will be needed to confirm the present results. 

### 4.2. HFUS

Early reports of ultrasound in CTCL date back to the late 1990s. However, until recently its clinical utility was limited by low-frequency transducers lacking adequate resolution [8,11,12,18,19]. 

A recently published Chinese study aimed to describe morphologic features of CTCL patch lesions using both 20 MHz and 50 MHz transducers. The authors described morphologic features, including the evenness of the epidermis and the presence of internal echoes and echogenic foci [12]. Mandava et al. published a retrospective multicenter study evaluating the ultrasound features of both cutaneous B-cell lymphoma and T-cell lymphoma patients, including data from four dedicated centers in India, Chile, Italy, and Spain [11]. While the authors found that CTCL plaques show irregular hypoechoic infiltrates, they concluded that the observed features are not exclusive for CTCL. This is in accordance with our study, were we found that morphologic features such as the evenness of the epidermis and the presence of internal echoes are too unspecific to distinguish CTCL-patch lesions from AD or CTCL plaques from psoriasis. 

Another way of evaluating ultrasound images in CTCL is by measuring the thickness of the skin. Polanska et al. studied the treatment response of three MF patients using an HFUS transducer of 20 MHz. They found that SLEB thickness decreased during phototherapy. The authors concluded that routine measurements of the SLEB thickness could serve as a good complementary assessment tool to monitor treatment response [8]. 

In our current study, we used an ultra-high frequency transducer with a maximum frequency of 70 Mhz. We measured the epidermal-, SLEB-, and dermal thickness and compared CTCL patients with AD and psoriasis, indicating increased epidermal thickness in CTCL and psoriasis compared with AD; however, our data are limited by the small sample size and warrant further large-scale studies from multiple participating sites to evaluate the clinical and diagnostic implication of skin ultrasound in CTCL. 

## Figures and Tables

**Table 1:** Dermoscopy features of cutaneous T-cell lymphomas (CTCL).

Dermoscopic Features		CTCL-Patch			CTCL-Plaque		Folliculotropic MF	
		Own Cases	Ghahramani et al. [13]	Lallas et al. [10]	Own Cases	Ghahramani et al. [13]	Own Cases	Ghahramani et al. [13]
**Vascular patterns**	Fine short linear vessels	2/4 (50%)	5/6 (83.3%)	30/32 (93.8%)	0/1	0/1	1/1 (100%)	2/5 (40%)
	Dotted vessels	2/4 (50%)	1/6 (16.7%)	18/32 (56.3%)	1/1 (100%)	1/1 (100%)	1/1 (100%)	2/5 (40%)
	Spermatozoa-like structures	2/4 (50%)	4/6 (66.7%)	16/32 (50.0%)	0/1	0/1	0/1	1/5 (20%)
	Pseudopod-like vessels	0/4	0/6	-	0/1	0/1	0/1	0/5
	Arborizing vessels	0/4	0/6	-	0/1	0/1	0/1	0/5
	Polymorphous vascular pattern	0/4	0/6	-	0/1	0/1	0/1	0/5
**Background**	Light red background	3/4 (75%)	1/6 (16.7%)	-	0/1	0/1	0/1	1/5 (20%)
	Dull red background	1/4 (25%)	3/6 (50%)	-	1/1 (100%)	1/1 (100%)	0/1	4/5 (80%)
**Scale**	White scale	3/4 (75%)	3/6 (50%)	6/32 (18.8%)	1/1 (100%)	1/1 (100%)	0/1	4/5 (80%)
	Yellow scale	0/4	0/6	0/32	0/1	0/1	0/1	0/5
	Structureless patches	2/4 (50%)	6/6 (100%)	-	0/1	1/1 (100%)	0/1	3/5 (60%)
**Other features**	Orange-yellowish patchy areas	0/4	0/6	29/32 (90.6%)	0/1	1/1 (100%)	0/1	0/5
	Crystalline structures	1/4 (25%)	2/6 (33.3%)	-	0/1	0/1	0/1	2/5 (40%)
	Yellow ulceration	0/4	0/6	-	0/1	0/1	0/1	1/5 (20%)
	Perifollicular accentuation	0/4	0/6	-	0/1	0/1	1/1 (100%)	5/5 (100%)
	Comedo openings	0/4	0/6	-	0/1	0/1	1/1 (100%)	3/5 (60%)

**Table 2:** A comparison of dermoscopy features seen in CTCL and atopic dermatitis (AD) and psoriasis patients.

Dermoscopy Features	CTCL-Patch (*n* = 42)	AD (*n* = 37)	*p*-Value	CTCL-Plaque (*n* = 2)	Psoriasis (*n* = 88)	*p*-Value
Fine short linear vessels	37/42	1/37	<0.001	0/2	2/88	1.0
Dotted vessels	21/42	32/37	0.001	2/2	88/88	n.a.
Spermatozoa-like structures	22/42	0/37	<0.001	0/2	0/5 *	n.a.
Pseudopod-like vessels	0/42	0/37	n.a.	0/2	0/5 *	n.a.
Arborizing vessels	0/42	0/37	n.a.	0/2	0/5 *	n.a.
Polymorphous vascular pattern	0/42	0/37	n.a.	0/2	0/5 *	n.a.
Light red background	4/10*	2/2*	0.46	0/2	37/88	0.51
Dull red background	4/10*	0/2*	0.52	2/2	51/88	0.51
White scale	12/42	25/37	0.001	2/2	65/88	1.0
Yellow scale	0/42	21/37	<0.001	0/2	3/88	1.0

The table represents pooled data from our own cases and data retrieved from the literature [10,13,14]. * Dermoscopic features were only assessed in our own patients and not assessed in previously published cases.

**Table 3:** High frequency ultrasound measurements.

Skin Thickness (µm)	CTCL-Patch (*n* = 5)	CTCL-Plaque (*n* = 1)	AD (*n* = 2)	Psoriasis (*n* = 4)	*p*-Value
Epidermis	271 ± 124	322	154 ± 32	215 ± 51	0.381
SLEB	193 ± 78	274	161 ± 55	168 ± 39	0.571
Dermis	1847 ± 460	1265	1663 ± 276	1900 ± 897	0.571
SLEB-Grade	3 (2–3)	3	2 (2)	3 (2–3)	n.a.

Mean ± standard deviation of epidermal-, SLEB- and dermal thickness in CTLCL-patch, CTCL plaque, atopic dermatitis and psoriasis as well as median (range) SLEB grade. Abbreviations: AD, atopic dermatitis; CTCL, cutaneous T-cell lymphoma; µm, micrometer; SLEB, subepidermal lower echogenic band.

## Data Availability

The data presented in this study are contained in the article tables and are available on request from the corresponding author.
